# Genomic Characterization of Carbapenemase-Producing *Klebsiella pneumoniae* ST895 Isolates from Canine Origins Through Whole-Genome Sequencing Analysis

**DOI:** 10.3390/microorganisms13020332

**Published:** 2025-02-03

**Authors:** Ronglei Huang, Wei Gao, Yue Sun, Yan Ye, Tingting Luo, Yitong Pan, Chengyang Zhang, Ang Zhou, Wenzhi Ren, Chongtao Du

**Affiliations:** 1 State Key Laboratory for Diagnosis and Treatment of Severe Zoonotic Infectious Diseases, Key Laboratory for Zoonosis Research of the Ministry of Education, Institute of Zoonosis, and College of Veterinary Medicine, Jilin University, Changchun 130062, China; hrl17835714615@163.com (R.H.); 13351590278@163.com (Y.Y.); mengda9969@163.com (Y.P.); zcy1123101929@163.com (C.Z.); 13290348002@163.com (A.Z.); 2 College of Animal Sciences, Jilin University, Changchun 130062, China; gaowei81@jlu.edu.cn (W.G.); 18847500911@139.com (Y.S.); 15590676136@163.com (T.L.); rwz1964@163.com (W.R.)

**Keywords:** beta-lactamase *NDM-5*, whole genome sequencing, *Klebsiella pneumoniae*, antimicrobial susceptibility test, veterinary medicine

## Abstract

The widespread application of carbapenems and other broad-spectrum antibiotics has significantly escalated the threat posed by highly drug-resistant *Klebsiella pneumoniae* to human public health. In this research, we isolated a carbapenem-resistant *K. pneumoniae* strain from the feces of pet dogs at a veterinary hospital in Changchun, Jilin Province, China. To gain insights into its genetic makeup and resistance mechanisms, we conducted comprehensive whole-genome sequencing and antimicrobial susceptibility testing on the isolated strain. Our findings revealed the presence of three distinct plasmids within the strain, classified as IncFIB&IncFII, IncR, and IncX3. Notably, the bla*_NDM-5_* gene, conferring resistance to carbapenems, was uniquely harbored on the IncX3 plasmid, which was devoid of any other resistance genes beyond bla*_NDM-5_*. In contrast, the remaining two plasmids, IncFIB&IncFII and IncR, were found to encode an array of additional drug resistance genes, contributing to the strain’s broad-spectrum resistance phenotype. The IncX3 plasmid, specifically, measures 45,829 bp in length and harbors the IS*5D*-bla*_NDM-5_*-Ble-MBL-PRAI cassette, which has been closely linked to the dissemination of bla*_NDM-5_* genes in *K. pneumoniae* strains. We reported the bla*_NDM-5_*-carrying IncX3 in *K. pneumoniae* isolates recovered from the pet dog and revealed the molecular characterization. Emphasis should be placed on, and continuous monitoring carried out for, the dissemination of *K. pneumoniae* harboring the bla*_NDM-5_* gene among humans, companion animals, and their related environments.

## 1. Introduction

*Klebsiella pneumoniae* is a clinically common Gram-negative opportunistic pathogen that can cause serious diseases such as pneumonia, pyogenic liver abscess, endophthalmitis, meningitis, and urinary tract infections in both humans and animals [1,2,3]. In recent years, due to the widespread use and abuse of broad-spectrum antibiotics such as carbapenems, the overlapping phenomenon of high virulence and high drug resistance of *K. pneumoniae* has attracted global attention, posing a huge threat to human public health [4,5,6].

Carbapenemase-producing Enterobacterales (CPE) has emerged as a pressing public health issue. The World Health Organization’s (WHO) latest Priority Pathogens List released in 2024 categorizes carbapenem-resistant Enterobacterales (CRE) as a Critical group, underscoring their urgency [5]. According to the China Antimicrobial Resistance Surveillance Network (CHINET; accessed on 15 July 2024, at https://www.chinets.com/), *K. pneumoniae* stands out as the second most prevalent clinical isolate, trailing closely behind *Escherichia coli*. Alarmingly, from 2005 to 2023, the resistance rates of *K. pneumoniae* to both imipenem and meropenem have consistently displayed a worrisome upward trajectory [7].

In the realm of veterinary medicine, the utilization of carbapenem antibiotics in animals is strictly prohibited; however, the challenge of managing multidrug-resistant (MDR) Gram-negative bacterial infections may prompt their exceptional prescription within the therapeutic cascade [8]. Globally, the incidence of CPE detection in animals has been steadily increasing, with companion animal-derived carbapenemase-producing *Escherichia coli* (CPEC) strains notably carrying the IncX3 plasmid that encodes for the bla*_NDM-5_* gene [9]. Furthermore, the gene that confers carbapenem resistance, known as the bla*_NDM_* gene, is primarily carried by plasmids, and its horizontal transfer is mediated by various plasmid types, which are often conjugative [10]. Given the scarcity of therapeutic alternatives for infections caused by these resistant bacteria, the potential for transmission of carbapenemase-producing bacteria from animals in close proximity to humans necessitates heightened vigilance and attention.

In this study, we conducted whole-genome sequencing on a *K. pneumoniae* strain isolated from a pet dog that harbors the bla*_NDM-5_* resistance gene. Using whole-genome phylogenetic analysis, we measured the genomic distances among all *K. pneumoniae* ST895 strains carrying the bla*_NDM-5_* resistance gene, regardless of their origins. Additionally, we performed linear alignments of the bla*_NDM-5_* loci among multiple *K. pneumoniae* strains that carry this resistance gene. To identify potentially undetected genes posing a threat, we screened and analyzed the whole-genome dataset against public databases.

## 2. Materials and Methods

### 2.1. Isolation, Purification, and Antimicrobial Susceptibility Testing of K. pneumoniae

A fecal sample from a female, three-year-old poodle at Dr. Xie’s Animal Hospital in Changchun, Jilin Province, was used to isolate and purify *K. pneumoniae* utilizing Brain Heart Infusion Broth (BHI) and SS agar media. The purified strain was then subjected to microscopic bacterial morphological observation and 16S rDNA sequencing for re-identification and confirmation, with the result confirming the presence of *K. pneumoniae*.

In accordance with the Performance Standards for Antimicrobial Susceptibility Testing published by the Clinical and Laboratory Standards Institute (CLSI) in 2022, the Kirby-Bauer (K-B) disk diffusion method was employed to observe and determine the susceptible of the isolated strain against a selection of 25 antibiotics [11]. The forceps were first sterilized, and sterile cotton swabs were prepared. A colony was picked and diluted with sterile physiological saline to prepare a bacterial suspension with a concentration of 0.5 McFarland standards. The diluted bacterial suspension was then applied to a Mueller-Hinton Agar plate using the sterile cotton swab. Sterilized forceps were used to place the antibiotic susceptibility disks at specified intervals across different locations on the agar plate. Following this, the plate was incubated in a bacterial incubator at 37 °C for 16–18 h. The resistance was determined according to the CLSI standards. Additionally, *K. pneumoniae* ATCC 700603 was selected as the quality control strain for the antimicrobial susceptibility test.

### 2.2. Whole-Genome Sequencing Analysis

The genomic DNA of *K. pneumoniae* strain FO528NT3 was sequenced by the Novogene Co., Ltd. (Beijing, China) using a NovaSeq 6000 System (Illumina Inc., San Diego, CA, USA) and a PacBio Sequel platform (PacBio, Menlo Park, CA, USA).

### 2.3. Genetic Analysis of the Bla_NDM-5_ Genes

We utilized the RAST server to predict and annotate the complete nucleotide genome sequence of *K. pneumoniae* FO528NT3. (https://rast.nmpdr.org/rast.cgi accessed on 24 November 2024) Antibiotic resistance genes in *K. pneumoniae* FO528NT3 were identified by querying the genome sequence against the ResFinder database (v.4.6.0). We identified the plasmid classification using PlasmidFinder 2.1 and introduced the sequence of *K. pneumoniae* FO528NT3 into MLST 2.0 for multilocus sequence typing (MLST) determination [12,13,14]. Comprehensive basic information regarding *K. pneumoniae* FO528NT3 was acquired through the aforementioned methods, encompassing the plasmids it harbors and their respective types, as well as the resistance genes located on both its chromosome and plasmids.

We utilized BLAST to compare plasmid sequences and employed BLAST Ring Image Generator (BRIG v.0.95) to identify and visualize the genomic circle map representing plasmid sequence comparisons. Furthermore, we then downloaded complete nucleotide sequences of several homologous plasmids carrying bla*_NDM-5_* in *K. pneumoniae* previously released at the NCBI website and comparison of the gene environment surrounding bla*_NDM-5_* gene was performed by Easyfig software (v.2.2.3) [15,16].

### 2.4. Phylogenetic Analysis

The reference genomes of representative sequence types from the phylogenetic analysis are listed in Supplementary Table S1, which also presents a graphical depiction based on the country of origin and the resistance genes harbored, as detailed in the Supplementary Information.

We screened 13 strains of ST895, 25 strains of ST11, and 17 strains of ST23 *K. pneumoniae* from the pathogen (https://pathogen.watch/ accessed on 28 November 2024) for phylogenetic analysis and the construction of a maximum likelihood phylogenetic tree involving *K. pneumoniae* strain FO528NT3. The single nucleotide polymorphism (SNP) alignment and maximum likelihood phylogenetic tree of isolates was performed and using Interactive Tree of Life (iTOL) version 6.0 in conjunction with heat mapping (https://itol.embl.de/ accessed on 29 November 2024).

### 2.5. Data Availability

The genomes of strain (FO528NT3) have been deposited in GenBank under BioProject number PRJNA1169760.

## 3. Results

### 3.1. Phenotyping of Drug Resistance of the Isolated Strain

The bla*_NDM-5_*-harboring *K. pneumoniae* isolate, FO528NT3, was discovered in the feces of a poodle at Dr. Xie’s Animal Hospital in Changchun City, Jilin Province, China. The results of the susceptible testing against 25 antibiotics revealed that the F0528NT3 exhibited resistance to a broad spectrum of antibiotics including gentamicin, penicillin, amikacin, ampicillin, ceftazidime, oxacillin, cefoperazone, cefazolin, piperacillin, cefuroxime, cephalexin, ceftriaxone, kanamycin, streptomycin, norfloxacin, ciprofloxacin, trimethoprim-sulfamethoxazole, colistin, and meropenem. Conversely, the isolate maintained susceptible to florfenicol, clindamycin, minocycline, lincomycin, levofloxacin, and tigecycline (Table S2).

### 3.2. Genetic Characterization of K. pneumoniae FO528NT3

**Figure 1:**
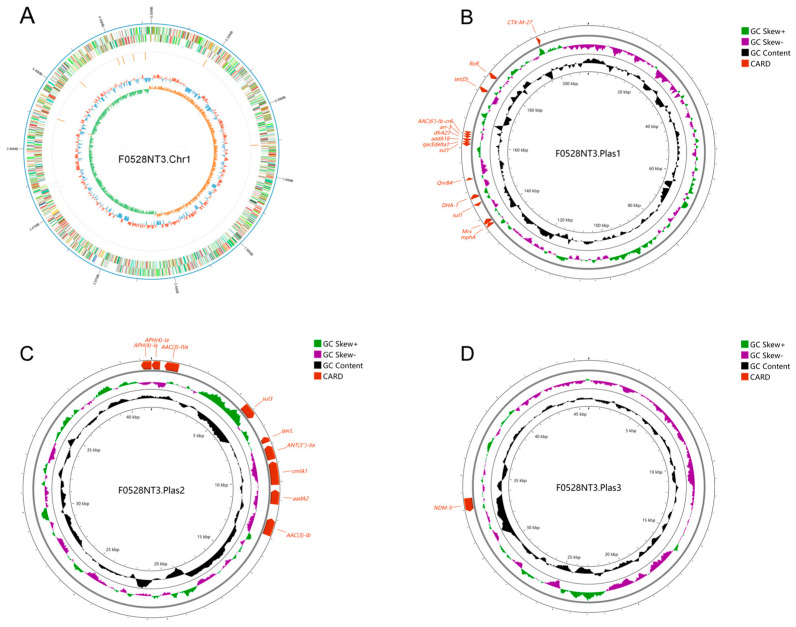
Genetic structure characteristics of the *K. pneumoniae* F0528NT3. (**A**) Genetic structural characteristics of the chromosome carried by *K. pneumoniae* F0528NT3; (**B**) Genetic structure characteristics of the pF0528NT3.Plas1 carried by *K. pneumoniae* F0528NT3; (**C**) Genetic structure characteristics of the pF0528NT3.Plas2 carried by *K. pneumoniae* F0528NT3; (**D**) Genetic structure characteristics of the pF0528NT3.Plas3 carried by *K. pneumoniae* F0528NT3. Resistance genes are coloured red.

Utilizing advanced third-generation whole-genome sequencing technology, *K. pneumoniae* FO528NT3 was comprehensively analyzed, revealing a single chromosomal sequence alongside three fully circularized plasmid sequences (Figure 1). The chromosome, spanning 5,260,035 base pairs (bp) in length, is assigned to MLST 895 and harbors an array of resistance genes, including bla*_SHV-182_*, *fosA6*, *OqxB*, and *OqxA*.

Plasmid pF0528NT3.Plas1, with a total length of 211,697 bp, exhibits a dual-replicon phenotype, belonging to both the IncFIB and IncFII types. This plasmid encapsulates a diverse array of resistance genes, such as bla*_CTX-M-27_*, *floR*, *tet(D)*, *AAC(6′)-Ib-cr6*, and bla*_DHA-1_*, further contributing to the strain’s antibiotic resistance profile. Then, Plasmid pF0528NT3.Plas2, measuring 41,604 bp in length, is classified as IncR type and carries resistance genes *APH(4)-Ia*, *AAC(3)-IVa*, *sul3*, and *qacL*, adding another layer of complexity to the strain’s resistance mechanisms. Lastly, Plasmid pF0528NT3.Plas3, with a total length of 45,829 bp, belongs to the IncX3 type and harbors the potent bla*_NDM-5_* resistance gene, emphasizing the strain’s capacity to resist even the most advanced antibiotics (Table S3).

Upon correlating the identified resistance genes with the observed resistance phenotypes, a strong concordance was found, indicating that the resistance genes present in *K. pneumoniae* FO528NT3 accurately reflect its resistance profile. The presence of three plasmids, each harboring multiple resistance genes, underscores the extensive drug resistance capabilities of this bacterial strain.

### 3.3. Phylogenetic Analysis of F0528NT3 K. pneumoniae Strain

**Figure 2:**
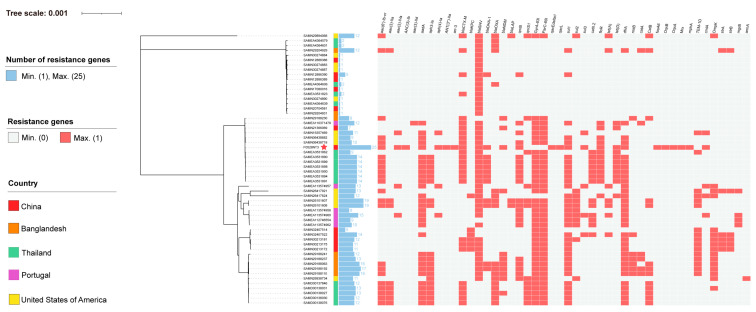
Phylogenetic analysis of *K. pneumoniae* ST895 isolates. Phylogenetic structure of *K. pneumoniae* maximum likelihood tree of ST895 clonotype *K. pneumoniae* isolates. The geographical location of the *K. pneumoniae* strains are indicated in different colors. The bar graphs indicate the number of resistance genes carried by each strain of *Klebsiella pneumonia.* The heat map indicate the resistance genes carried by each strain of *Klebsiella pneumonia.* The strain marked with the red five-pointed star is F0528NT3, which originates from this study.

To elucidate the phylogenetic relationships and evolutionary trajectories among the ST895 *K. pneumoniae* isolate F0528NT3 from our study, in comparison to other ST895 strains and the predominant serotypes ST11 and ST23 of *K. pneumoniae*, we constructed a maximum likelihood phylogenetic tree (Figure 2). Upon conducting an exhaustive database search, we identified a collective thirteen additional ST895 *K. pneumoniae* strains, originating from geographically dispersed locations: seven from Thailand, three from the United States of America, one from China, one from Bangladesh, and one from Portugal (accessible at https://pathogen.watch/ accessed on 28 November 2024). Our comprehensive analysis demonstrated that F0528NT3 shares a substantial degree of homology with ST895 *K. pneumoniae* strains isolated from diverse sources, encompassing human blood, human tissue, human pus, and feces. Notably, within the context of the major prevalent serotypes ST11 and ST23, ST895, which encompasses F0528NT3, demonstrates a closer phylogenetic affinity to ST11. Furthermore, both ST895 and ST11 strains are characterized by the prevalent carriage of multiple drug resistance genes (Table S1).

Additionally, it is important to highlight that all 13 ST895 *K. pneumoniae* strains identified in our analysis possess β-lactamase resistance genes, including bla*_DHA-1_*, bla*_SHV-11_*, or bla*_CTX-M-15_*. These strains also exhibit a diverse array of additional antibiotic resistance genes, underscoring their capacity for multidrug resistance.

### 3.4. Gene Function Analysis of K. pneumoniae FO528NT3

**Figure 3:**
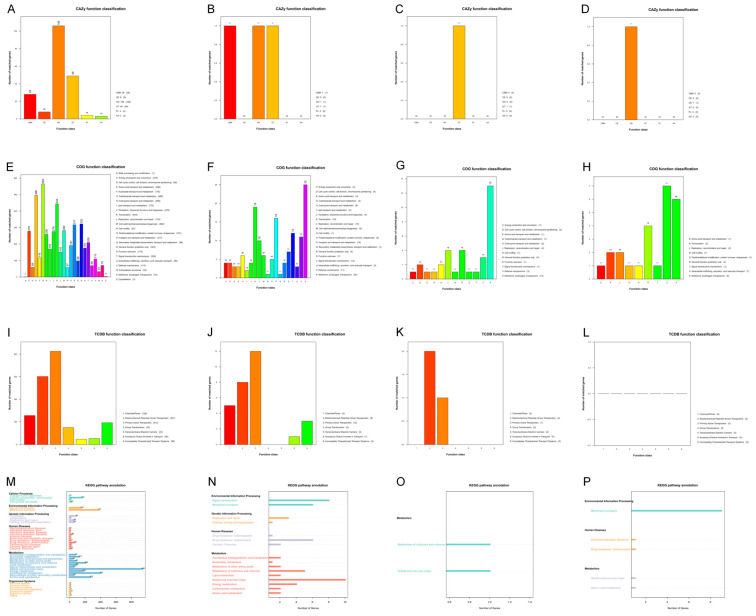
The annotation of functional genes in *K. pneumoniae* FO528NT3. (**A**) The Carbohydrate-Active enZYmes Database (CAZy) function classification of the chromosome in *K. pneumoniae* FO528NT3; (**B**) The CAZy function classification of pF0528NT3.Plas1 carried by *K. pneumoniae* F0528NT3; (**C**) The CAZy function classification of pF0528NT3.Plas2 carried by *K. pneumoniae* F0528NT3; (**D**) The CAZy function classification of pF0528NT3.Plas3 carried by *K. pneumoniae* F0528NT3; (**E**) The Cluster of Orthologous Groups of proteins (COG) function classification of the chromosome in *K. pneumoniae* FO528NT3; (**F**) The COG function classification of pF0528NT3.Plas1 carried by *K. pneumoniae* F0528NT3; (**G**) The COG function classification of pF0528NT3.Plas2 carried by *K. pneumoniae* F0528NT3; (**H**) The COG function classification of pF0528NT3.Plas3 carried by *K. pneumoniae* F0528NT3; (**I**) The Transporter Classification Database (TCDB) function classification of the chromosome in *K. pneumoniae* FO528NT3; (**J**) The TCDB function classification of pF0528NT3.Plas1 carried by *K. pneumoniae* F0528NT3; (**K**) The TCDB function classification of pF0528NT3.Plas2 carried by *K. pneumoniae* F0528NT3; (**L**) The TCDB function classification of pF0528NT3.Plas3 carried by *K. pneumoniae* F0528NT3; (**M**) The Kyoto Encyclopedia of Genes and Genomes (KEGG) pathway annotation of the chromosome in *K. pneumoniae* FO528NT3; (**N**) The KEGG pathway annotation of pF0528NT3.Plas1 carried by *K. pneumoniae* F0528NT3; (**O**) The KEGG pathway annotation of pF0528NT3.Plas2 carried by *K. pneumoniae* F0528NT3; (**P**) The KEGG pathway annotation of pF0528NT3.Plas3 carried by *K. pneumoniae* F0528NT3.

Based on third-generation whole-genome sequencing technology, the assembly of *K. pneumoniae* FO528NT3 yielded one chromosomal sequence and three complete circular plasmid sequences, which were subsequently annotated for their genes. In the Carbohydrate-Active enZYmes Database (CAZy), the chromosome harbored the vast majority of relevant carbohydrate-active enzymes, with Glycoside Hydrolases (GHs) accounting for 53.54% (106/198) of the total (Figure 3A). Plasmid pF0528NT3.Plas1 contained three related carbohydrate-active enzymes (Figure 3B), while plasmids pF0528NT3.Plas2 and pF0528NT3.Plas3 each harbored one (Figure 3C,D). Within the Cluster of Orthologous Groups of proteins (COG), the chromosome aligned to 24 protein sequence categories, with those related to carbohydrate transport and metabolism being the most abundant (11.89%, 565/4752). There were 70 protein sequences associated with Mobilome: prophages, transposons (Figure 3E). Plasmids pF0528NT3.Plas1 and pF0528NT3.Plas2 had the highest number of protein sequences related to Mobilome: prophages, transposons, with 25 and 13, respectively (Figure 3F,G). Plasmid pF0528NT3.Plas3 had the most protein sequences related to Intracellular trafficking, secretion, and vesicular transport (28.00%, 7/25), and contained six protein sequences associated with Mobilome: prophages, transposons (Figure 3H). In the Transporter Classification Database (TCDB), the chromosome and plasmid pF0528NT3.Plas1 predominantly featured proteins related to Primary Active Transporter, with 413 and 12, respectively (Figure 3I,J). Plasmid pF0528NT3.Plas2 had the most proteins related to Electrochemical Potential-driven Transport (66.67%, 2/3) (Figure 3K). No relevant proteins were identified in plasmid pF0528NT3.Plas3 (Figure 3L). In the Kyoto Encyclopedia of Genes and Genomes (KEGG), the chromosomes, plasmid pF0528NT3.Plas1, and plasmid pF0528NT3.Plas2 each contained the highest number of genes related to Metabolism in the Global and overview maps (Figure 3M–O). Plasmid pF0528NT3.Plas3 primarily contained genes related to membrane transport (Figure 3P).

**Figure 4:**
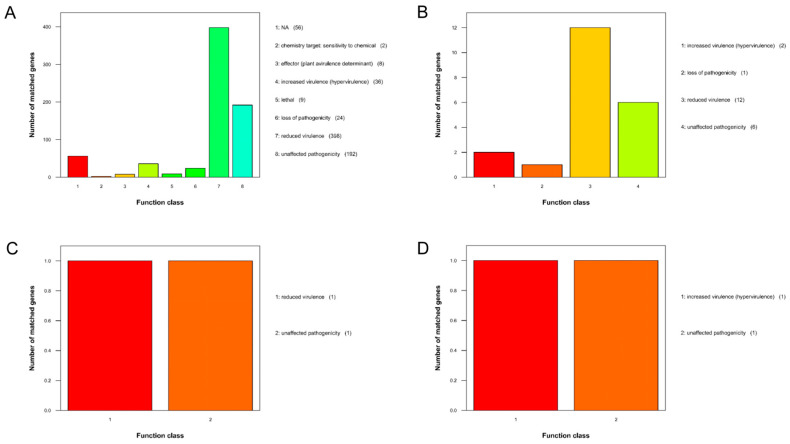
Annotation in the Pathogen-Host Interactions database (PHI) for *K. pneumoniae* FO528NT3. (**A**) The PHI phenotype classification of the chromosome in *K. pneumoniae* FO528NT3; (**B**) The PHI phenotype classification of pF0528NT3.Plas1 carried by *K. pneumoniae* F0528NT3; (**C**) The PHI phenotype classification of pF0528NT3.Plas2 carried by *K. pneumoniae* F0528NT3; (**D**) The PHI phenotype classification of pF0528NT3.Plas3 carried by *K. pneumoniae* F0528NT3.

In the Pathogen Host Interactions Database (PHI), the chromosome, plasmid pF0528NT3.Plas1, and plasmid pF0528NT3.Plas2 mainly harbored genes with mutant phenotypes related to reduced virulence, with counts of 398, 12, and 1, respectively (Figure 4A–C). Notably, plasmid pF0528NT3.Plas3 contained one gene (50%, 1/2) with a mutant phenotype related to increased virulence (hypervirulence) (Figure 4D).

### 3.5. Genetic Structure Characteristics of Plasmid Carrying Bla_NDM-5_ Resistance Gene

**Figure 5:**
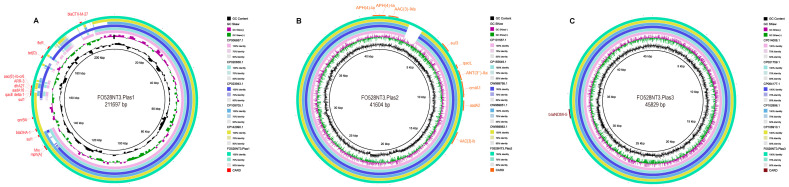
The circular comparison diagram of plasmid genomes carried by *K. pneumoniae* FO528NT3. (**A**) In comparing plasmid pF0528NT3.Plas1 with other similar plasmids, it is noted that pF0528NT3.Plas1 serves as the reference plasmid. Each circle, from the innermost to the outermost, represents a different plasmid as follows: CP006657.1, CP020838.1, CP023943.1, CP109705.1, CP142990.1 and pF0528NT3.Plas1 (this study). (**B**) In comparing plasmid pF0528NT3.Plas1 with other similar plasmids, it is noted that pF0528NT3.Plas2 serves as the reference plasmid. Each circle, from the innermost to the outermost, represents a different plasmid as follows: CP101557.1, CP155649.1, OW969795.1, OW969851.1, OW969863.1 and pF0528NT3.Plas2 (this study). (**C**) Comparison of bla*_NDM-5_*-carrying plasmid pF0528NT3.Plas3 with other similar plasmids. Plasmid pF0528NT3.Plas1 is a reference plasmid. Each circle represents a different plasmid as follows (from inside to outside): CP014006.1, CP021759.1, CP064177.1, CP102896.1, CP106910.1 and pF0528NT3.Plas3 (this study).

Upon comparative analysis of the three plasmids harbored by *K. pneumoniae* FO528NT3, it was revealed that plasmid F0528NT3.Plas1 not only shares a substantial number of drug resistance genes with comparable plasmids but also possesses unique resistance genes, namely bla*_DHA-1_*, *qnrB1*, and *tet(D)* (Figure 5A). Conversely, plasmid F0528NT3.Plas2 exhibits identical drug resistance genes with five similar plasmids (Figure 5B). Notably, plasmid F0528NT3.Plas3 solely carries the bla*_NDM-5_* resistance gene, devoid of any other resistance genes on its sequence. With the sequence of this latter plasmid serving as the reference, all five selected plasmids demonstrated sequences that align with the entire genomic information of F0528NT3.Plas3, encompassing the bla*_NDM-5_* gene (Figure 5C).

**Figure 6:**
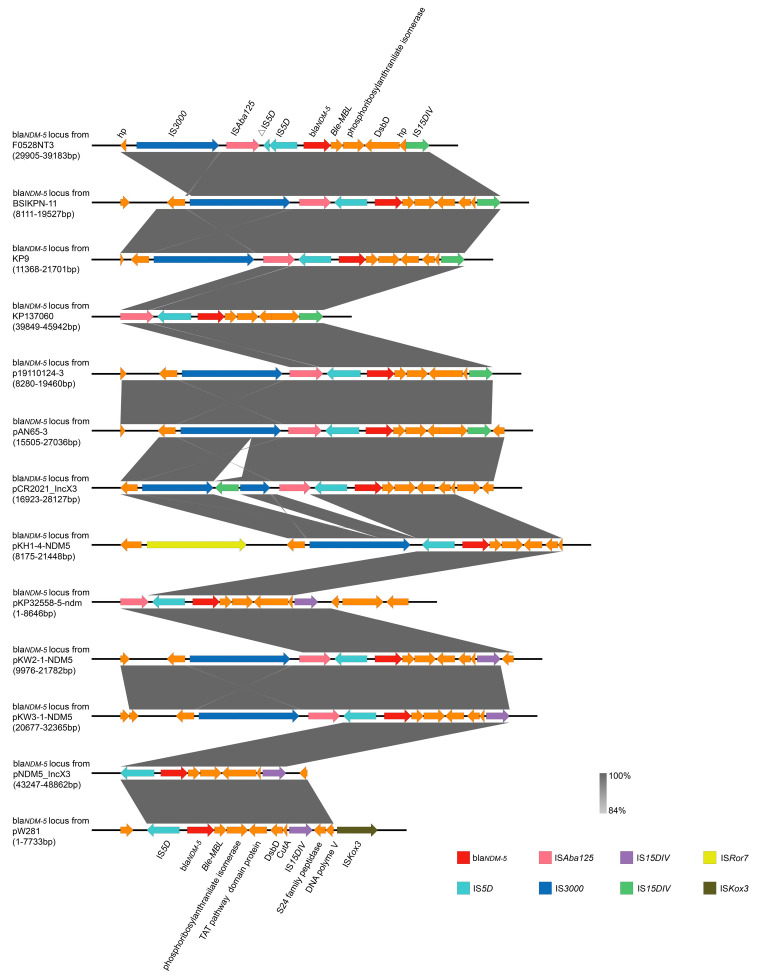
Genetic environment of bla*_NDM-5_* in *K. pneumoniae*. Comparative sequence analysis of regions of bla*_NDM-5_* of pF0528NT3.Plas3 with other plasmid structures harboring bla*_NDM-5_*. Genes are denoted by arrows. Resistance genes are coloured red. Mobile elements and other features are coloured red, green, purple, etc. Shading denotes regions of homology (nucleotide identity ≥ 84%).

Upon conducting a thorough search of the NCBI database for *K. pneumoniae* strains harboring the bla*_NDM-5_* resistant gene (Table S4), we performed a detailed linear alignment of the upstream and downstream flanking regions of the bla*_NDM-5_* gene in 12 such strains with the corresponding regions of the bla*_NDM-5_* gene locus in pF0528NT3.Plas3 (Figure 6). Our analysis revealed that the IS*5D*-bla*_NDM-5_*-Ble-MBL-PRAI gene cassette is remarkably conserved among *K. pneumoniae* strains. Furthermore, we observed a recurrent presence of IS*Aba125* and IS*3000* insertion sequences upstream of this conserved gene cassette, highlighting their potential role in the dissemination and stability of the bla*_NDM-5_* resistance determinant.

## 4. Discussion

In recent years, the emergence of carbapenem resistance in Enterobacteriaceae has attracted significant global attention, with *K. pneumoniae* showing an overall upward trend in resistance rates to imipenem and meropenem. According to statistics, in 2019 alone, carbapenem-resistant pathogens (such as *Acinetobacter baumannii* and *K. pneumoniae*) led to the deaths of 50,000 to 100,000 people worldwide [17]. An increasing number of studies have reported the detection of carbapenem-resistant *K. pneumoniae* in companion animals, the environment, and humans [18,19,20,21]. In this study, we detected a bla*_NDM_*-positive *K. pneumoniae* isolate from the feces of companion animals, highlighting the potential threat to the safety of companion animals as well as humans.

Carbapenem antibiotics and colistin are considered the “last line of defense” antibiotics [22]. However, studies have found that plasmids (pX3_NDM-5) carrying the bla*_NDM-5_* encoding gene have a broad host range and can be transferred among up to 16 bacterial phyla, rather than the previously believed narrow host range. Furthermore, Gram-negative bacteria can transfer pX3_NDM-5 to Gram-positive bacteria, and this plasmid can also be retransferred back to Gram-negative bacteria [23]. In this study, the IS*5D*-bla*_NDM-5_*-Ble-MBL-PRAI gene fragment was found to be highly conserved in *K. pneumoniae*, and upstream of this gene fragment, IS*Aba125* and IS*3000* insertion sequences often exist. Additionally, the IncX3 plasmid carrying the bla*_NDM-5_* gene in this study lacks a conjugative transfer region, which may contribute to the clonal spread of ST895 *K. pneumoniae* carrying bla*_NDM-5_*.

In the research conducted by Zhao et al., several *K. pneumoniae* strains bearing uncommon sequence types (ST690, ST895, ST1823, and ST1384) were identified within a major Eastern Chinese medical center, spanning the years from 2003 to 2016 [24]. Furthermore, it was found that ST11 and ST895 belonged to the same clonal complex. In this study, 14 strains of ST895 *K. pneumoniae* exhibited close phylogenetic relationships with ST11 *K. pneumoniae*, consistent with the findings reported by Zhao et al. within the research.

The study revealed that all CPEC strains originating from companion animals harbored the IncX3 plasmid, which encodes the bla*_NDM-5_* gene [25]. Similarly, the *K. pneumoniae* isolate in this study also contained the IncX3 plasmid, with the bla*_NDM-5_* gene being encoded by this plasmid. This indicates that the IncX3 plasmid has a preference for the dissemination of the bla*_NDM-5_* gene. Notably, within the context of the major prevalent serotypes ST11 and ST23, ST895, which encompasses F0528NT3, demonstrates a closer phylogenetic affinity to ST11. Furthermore, both ST895 and ST11 strains are characterized by the prevalent carriage of multiple drug resistance genes. Through comparative analyses using the CAZy, COG, KEGG, TCDB, and PHI databases, it has been observed that plasmids harboring a greater number of antibiotic resistance genes exhibit an increased abundance of protein sequences associated with Mobilome components, specifically prophages and transposons. Furthermore, plasmids pF0528NT3.Plas1 and pF0528NT3.Plas2 predominantly carry genes mutated in pathogen PHI phenotypes that are linked to reduced virulence. Conversely, plasmid pF0528NT3.Plas3, which carries the bla*_NDM-5_* gene, predominantly harbors genes mutated in pathogen PHI phenotypes associated with increased virulence, or hypervirulence. Consequently, the spread of these strains should be taken seriously, as they are capable of not only causing infections but also resisting a wide range of antimicrobial agents.

## 5. Conclusions

In conclusion, our comprehensive whole-genome analysis has unveiled the genomic capabilities of *K. pneumoniae* isolates harboring the bla*_NDM-5_* gene, sourced from companion animals. The findings underscore that these isolates frequently encode a multitude of drug resistance genes, encompassing β-lactam, aminoglycoside, and tetracycline resistance genes, with a subset additionally possessing carbapenemase resistance genes. Consequently, they pose a significant potential risk to public health. Given this scenario, it is imperative to broaden our focus beyond the well-documented high-virulence and multidrug-resistant strains such as ST11 and ST23, and to consider implementing novel strategies to curb the dissemination of less common yet highly resistant *K. pneumoniae* strains like ST895 within our society. This endeavor necessitates an integrated approach that harmonizes public health methodologies with the “One Health” perspective.

## Data Availability

Data is contained within the article or Supplementary Material.

## Supplementary Materials

The following supporting information can be downloaded at: https://www.mdpi.com/article/10.3390/microorganisms13020332/s1, Table S1: Sample accession; Table S2: The phenotypic resistance profile of *K. pneumoniae* FO528NT3; Table S3: Whole Genome Sequencing (WGS) data of the FO528NT3 *K. pneumoniae* isolate; Table S4: General features of the 13 bla_NDM-5_-harbouring plasmids.
