# Supplementary material for: Genomic Characterization of Carbapenemase-Producing Klebsiella pneumoniae ST895 Isolates from Canine Origins Through Whole-Genome Sequencing Analysis
Source: Microorganisms. 2025 Feb 3;13(2):332. doi: 10.3390/microorganisms13020332 (PMC11858644; doi:10.3390/microorganisms13020332)
Supplement: Supplementary file 1 [file microorganisms-13-00332-s001.zip › Supplementary Materials Table S2.pdf]

Table S2. The phenotypic resistance profile of *Klebsiella pneumoniae* FO528NT3.

Antimicrobialagent	antimicrobial susceptibility
gentamicin	R
amikacin	R
kanamycin	R
streptomycin	R
penicillin	R
ampicillin	R
oxacillin	R
ceftazidime	R
cefoperazone	R
cefazolin	R
piperacillin	R
cefuroxime	R
cephalexin	R
ceftriaxone	R
norfloxacin	R
ciprofloxacin	R
levofloxacin	S
trimethoprim-sulfamethoxazole	R
colistin	R
meropenem	R
florfenicol	S
clindamycin	S
minocycline	S
lincomycin	S
tigecycline	S
