# Supplementary material for: Genomic Characterization of Carbapenemase-Producing Klebsiella pneumoniae ST895 Isolates from Canine Origins Through Whole-Genome Sequencing Analysis
Source: Microorganisms. 2025 Feb 3;13(2):332. doi: 10.3390/microorganisms13020332 (PMC11858644; doi:10.3390/microorganisms13020332)
Supplement: Supplementary file 1 [file microorganisms-13-00332-s001.zip › Supplementary Materials Table S3.pdf]

Table S3. Whole Genome Sequencing (WGS) data of the FO528NT3 *K. pneumoniae* isolate.

Genome ID	Sequence Type	plasmid Type	Resistance Genes
F0528NT3.chr1	895	/	bla*SHV-182*、*fosA6*、*OqxB*、*OqxA*
pF0528NT3.Plas1	/	IncFIB&IncFII	bla*CTX-M-27*、*floR*、*tet(D)*、*AAC(6')-Ib-cr6*、*arr-3*、*dfrA27*、*aadA16*、*qacEdelta1*、*sul1*、*QnrB4*、*blaDHA-1*、*Mrx*、*mphA*
pF0528NT3.Plas2	/	IncR	*APH(4)-Ia*、*AAC(3)-IVa*、*sul3*、*qacL*、*ANT(3'')-IIa*、*cmlA1*、*aadA2*、*AAC(3)-Ib*
pF0528NT3.Plas3	/	IncX3	bla*NDM-5*
