# Supplementary material for: Genomic Characterization of Carbapenemase-Producing Klebsiella pneumoniae ST895 Isolates from Canine Origins Through Whole-Genome Sequencing Analysis
Source: Microorganisms. 2025 Feb 3;13(2):332. doi: 10.3390/microorganisms13020332 (PMC11858644; doi:10.3390/microorganisms13020332)
Supplement: Supplementary file 1 [file microorganisms-13-00332-s001.zip › Supplementary Materials Table S4.pdf]

Table S4. General features of the 13 bla$_{NDM-5}$-harbouring plasmids.

Plasmid	GenBank accession	Total length (bp)	Host bacterium	isolation source	Location
pF0528NT3.Plas3	CP178614	45829	*Klebsiella pneumoniae*	feces	China
BSIKPN-11unnamed4	CP106910	46161	*Klebsiella pneumoniae*	blood	China
KP9 plasmid unnamed3	CP130677	46162	*Klebsiella pneumoniae*	NA	China
KP137060 plasmid unnamed1	MW218142	46161	*Klebsiella pneumoniae*	sputum	China
p19110124-3	CP064177	46161	*Klebsiella pneumoniae*	Anal swab	China
pAN65-3	MK317995	46161	*Klebsiella pneumoniae*	feces	China
pCR2021_IncX3	CP147871	55817	*Klebsiella pneumoniae*	NA	China
pKH1-4-NDM5	CP102881	52085	*Klebsiella pneumoniae*	NA	China
pKP32558-5-ndm5	CP076035	46161	*Klebsiella pneumoniae*	balf	China
pKW2-1-NDM5	CP102890	46161	*Klebsiella pneumoniae*	well water	China
pKW3-1-NDM5	CP102892	46161	*Klebsiella pneumoniae*	well water	China
pNDM5_IncX3	CP065346	49941	*Klebsiella pneumoniae*	river	China
pW281_2	CP162994	45237	*Klebsiella pneumoniae*	rectal wipes	China

Note: All the completely sequenced and nonredundant bla_{NDM-5}-carrying plasmids available in GenBank (last accessed 1 September 2024) are included. NA, not applicable.
